# Transfusion practice in orthotopic liver transplantation

**DOI:** 10.4103/0972-5229.58536

**Published:** 2009

**Authors:** Allanki Surekha Devi

**Affiliations:** **From:** Department of Transfusion Medicine, Global Hospitals, Hyderabad - 500 004, India

**Keywords:** Blood loss, factors, liver transplantation, transfusion

## Abstract

Liver transplant procedures require the most blood components, despite the fact that blood use in liver transplantation has declined dramatically over the last decade. Liver transplant recipients present unique challenges, not only in terms of blood supply, but also requirements for specialized blood components, serologic problems, and immunologic effects of transfusion on both the allograft and the recipient. The cause of intraoperative blood loss in liver transplantation is multifactorial, due to both technical factors and poor coagulation control. This procedure carries the risk of massive blood loss, which requires massive transfusions and is associated with postoperative infections, reduced graft survival, multi-organ dysfunction, and higher risk of mortality. Efforts to reduce intraoperative bleeding leading to limitation of blood transfusions are desirable to improve results and also to control costs.

Method of literature search:

1. The name of topic is typed and searched in Google search.
2. The name of topic is typed and searched in PubMed search. Related articles were also searched.
3. Some standard books in Transfusion Medicine were also referred.

## Introduction

Liver transplantation is the treatment of choice in patients with acute or chronic end stage liver disease (ESLD), irresectable primary liver tumors, and metabolic disorders.[1] Orthotopic liver transplantation (OLT) is the replacement of a diseased liver with a healthy liver in the normal anatomic position. The operative procedure is extensive, complex, and technically challenging with multiple vascular transections and anastomoses.[2] In addition, OLT is associated with several hemostatic defects that contribute to a risk of massive blood loss.[2] The liver is an extremely vascular organ. The associated coagulopathy, anemia, malnutrition, and severe portal hypertension have made this procedure more daunting and the use of blood products is almost universal.[3] Hence, of all solid organ transplantations, OLT has placed the greatest demands on clinical transfusion services.[4] Although blood use has steadily declined with improved surgical and anesthesiological techniques, better graft preservation, better intraoperative monitoring of coagulation status, and pharmacologic treatment of fibrinolysis during the last decade, OLT still frequently demands transfusion equal to one blood volume (massive transfusion).[5] Because the transfusion needs during OLT are unpredictable, transfusion services must remain prepared to effectively deliver massive transfusion support. Liver transplant recipients present unique challenges, not only in terms of blood supply, but requirements of specialized blood components, serologic problems, and immunologic effects of transfusion on both the allograft and the recipient.[6] Studies have observed that increased blood requirements are associated with higher incidence of infections, drug overdose, prolonged stay in the ICU, serologic problems, and immunologic effects of transfusion on both the allograft and the recipient, graft rejection or graft death and patient death.[1–6] However, whether these differences in outcomes are related to the transfusion as an independent risk factor, or whether the transfusion is a marker for a technically more difficult surgery remains unclear. Yet, bloodless liver transplantation has been achieved in Jehovah's witness patients.[2] This subset of patients has allowed us a distinctive opportunity to develop strategies towards a transfusion-free environment, with ultimate aim of being able to translate this practice to all general surgical procedures.[3]

Donor leucocyte microchimerism is under active investigation as a mechanism to improve graft survival.[7] Starzl *et al*., proposed that the long-term presence of donor leucocytes may lead to tolerance of the graft.[7] Studies have shown that patients receiving cadaveric marrow stem cells at the time of liver transplantation had fewer rejection episodes and better graft survival than control patients.[7]

Method of literature search:

1. The name of topic is typed and searched in Google search.
2. The name of topic is typed and searched in PubMed search; went through the related articles.
3. Some standard books in Transfusion Medicine were also referred.

Contributing factors to blood loss during liver transplantation can be categorized based on factors related to preoperative and intraoperative factors.

## Transfusion Predictors

### Preoperative factors

Preoperative factors associated with blood loss during OLT include liver failure, cirrhosis, cholestasis, and splenomegaly.[2] The liver plays a central role in hemostasis. Many complex derangements of hemostasis are associated with ESLD which are shown in Table 1.[1]

**Table 1:** Hemostatic abnormalities in liver disease

Hypocoagulability
Deficiency of coagulation factors by impaired synthesis
Synthesis of abnormal clotting proteins (dysfibrinogenemia)
Impaired clearance of activated coagulation factors and degradated fibrin
Vitamin K deficiency
Hypercoagulability
Decreased levels of antithrombin, protein C or protein S by impaired synthesis
Enhanced fibrinolytic activity
Increased levels of circulating t-PA by impaired hepatic clearance
Reduced synthesis of fibrinolytic inhibitors
Quantitative and qualitative platelet defects
Splenomegaly caused by portal hypertension leads to platelet sequestration and destruction
Thrombopoietin deficiency due to cirrhosis leads to low platelet production
Disturbed platelet-vessel wall interaction
Inhibition of GP IIb/IIIa by increased levels of fibrin degradation products
Degraded platelet receptors by increase in plasmin levels
Disseminated intravascular coagulation
Consumption of coagulation factors and platelets
Hyperfibrinolysis
Impaired platelet function due to fibrin degradation products, secondary to hyperfibrinolysis

Two scoring systems have been used to grade the severity of ESLD and prioritize the patients on waiting list for OLT.

## Child-Turcotte-Pugh Score

CTP score is a measure of disease severity and is classified into CTP class A to C with a scoring system of 5 to 15.[2]

Class A: 5-6 (less severe)

Class B: 7-9

Class C: 10-15 (more severe)

### Model for end-stage liver disease

MELD, a numerical scale, ranging from 6 (less ill) to 40 (gravely ill), used for liver transplant patients gives a score based on how urgently the patient needs a liver transplant within the next three months. Its impact on transfusion requirements can be translated directly to the timing of the transplantation. With longer waiting times the liver disease progresses, as reflected in higher Child-Pugh/MELD score and thereby increasing transfusion requirement.

### Etiology of liver disease

Patients with chronic active hepatitis have more advanced disease and require more blood products than patients with primary biliary cirrhosis.[2]

### Preoperative hematological parameters

A retrospective study of 300 liver transplants reported no correlation among preoperative platelet count, aPTT, PT, fibrinogen to that of intraoperative blood loss or transfusion requirements.[2] Blood transfusions in OLT should be based on clinical assessment and well established transfusion protocol rather than laboratory tests.[2]

### Previous abdominal surgery

Those with previous upper abdominal surgery tend to have vascularised adhesions which may render liver dissection hemorrhagic thereby increasing intraoperative transfusion requirement.[2]

### Transjugular intrahepatic porto systemic shunt

TIPSS is done in patients with liver disease for intractable variceal bleed and refractory ascites. The benefits noted were that it decreases the bleeding during surgery by virtue of lowered portal pressures thereby decreasing blood requirements.[2]

### Preexisting coagulopathy

It is associated with increased blood loss during surgery, thereby increasing blood transfusions.[2]

### Intra-operative factors

Liver transplantation may be divided into three stages:

Stage I (preanhepatic phase): Patient's diseased liver and its vessels are dissected free and ends with removal of the diseased organ. Blood loss occurs from transection of collaterals that develops from portal hypertension. Preexisting abnormalities of clotting, platelets, and fibrinolysis compound the problem.[4]

Stage II (anhepatic phase): Begins with implantation of the donor liver and ends with graft reperfusion. This is a dangerous moment in the procedure when hemodynamic, metabolic, and hemostatic abnormalities can arise.[4]

Stage III (postreperfusion phase): Begins with reperfusion of the grafted liver, creating hepatic arterial anastamosis, preparing a form of biliary drainage for the new liver, obtaining good surgical hemostasis and closing.[4]

During surgery, marked changes in coagulation can occur. These may result from hemodilution, platelet consumption, disordered thrombin regulation, and fibrinolysis. Some patients develop severe coagulopathy, during the anhepatic and early reperfusion phase of OLT.[4] Transplantation of a healthy liver restores the patient's clotting function. However, a dysfunctional graft may not immediately produce clotting factors, thereby leading to coagulopathy mandating massive transfusions.[2]

#### Thromboelastography:

Besides standard coagulation tests (i.e., PT, aPTT, fibrinogen levels), TEG is a measurement technique allowing rapid on-site assessment of the functional clotting status.[8] TEG findings have been correlated with intraoperative hemorrhage and coagulopathy and can assist anesthesiologists in treating intraoperative bleeding by identifying the cause.[2] In combination with clinical bleeding assessment, it facilitates selective use of component therapy and specific drug treatment.[8]

#### Graft quality:

Steatosis increases cold ischemic injury and reduces the rate of hepatic regeneration.[9] The risk of primary nonfunction after transplantation of cadaveric graft increases proportionately with the degree of steatosis.[9] Marcos *et al*., suggested that steatosis reduces the percentage of the functioning liver.[9] Increased risk of graft dysfunction is observed in fatty infiltration of >30%, abnormal liver tests and other donor risk factors such as high inotrope administration and donor stay in the intensive care unit (>five days).[10] Graft dysfunction further necessitates massive transfusions.

Older donors (>60 years) are vulnerable to prolonged cold ischemia and high inotrope levels, give rise to early graft dysfunction and prolonged cholestasis.[10] Such recipients are vulnerable to receive massive transfusions.

#### Graft-recipient body weight ratio:

GRWR <0.8% in partial graft transplants (living donor liver transplants and split cadaveric transplants) leads to small-for-size syndrome (SFSS) which results in lower graft survival.[10] Such small-for size grafts depend on appropriate blood component therapy until the graft regenerates. A GRWR ratio of 1% is accepted to be a graft of good size.[9]

#### Graft preservation:

University of Wisconsin (UW) solution and Histidine-tryptophan-ketogluterate (HTK) solution are used as the primary preservation solution for liver allograft.[11] Improper and suboptimal graft preservation leads to graft dysfunction and to severe post transplant bleeding because of failure to synthesize coagulation factors which accelerates massive and multiple blood transfusions.

#### Cold ischemia time:

Length of CIT is the length of time an organ is preserved between procurement (organ recovery) and transplantation. Prolonged CIT continues to have a negative effect on liver transplant outcomes.[12] CIT had a significant effect on short-term graft failure.[12] CIT should be kept as short as possible to reduce the intraoperative RBC transfusion requirement as prolonged CIT has a negative effect on perioperative RBC transfusion requirement.[13]

#### Surgical technique:

OLT involves explantation of native liver and replacement with the donor liver. This is done by piggyback transplantation, whereby the inferior vena cava is preserved and venovenous bypass is avoided.[2] Portosystemic shunting is done in patients with liver failure in order to decrease preoperative complications associated with portal hypertension (for example, bleeding varices, ascites, sepsis). This decreases blood requirement and operative time.[2] The experience of the surgical and anaesthesiology team, impacts blood loss and transfusion requirement. Additionally, modifications in surgical technique, including use of electrocautery, argon beam coagulation and use of fibrin glue, minimizes blood loss thereby minimizing transfusions.[2] Meticulous surgical technique and securing hemostasis on the operating table remains a priority for the surgeon, and indeed long operating times translate into higher transfusion requirement.

#### Blood loss:

Blood loss is directly proportional to the duration of surgery. Consumption of banked blood (number of RBC units) reflects the degree of blood loss. Based on the consumption of RBC units, patients are categorized into High Blood Loss (HBL) group (>6 RBC units transfused) and Low Blood Loss (LBL) group (<6 RBC units transfused).[14] Intraoperative bleeding remains a significant problem affecting the immediate outcome after transplantation of liver.[15] Preoperative parameters cannot predict operative bleeding accurately and the mainstay to prevent bleeding is a meticulous surgical technique during the hepatectomy and correction of coagulation abnormalities throughout the procedure.[15]

#### Hemodynamics:

Central venous pressure (CVP) monitoring is an important aspect of OLT. Patients undergo volume expansion prior to hepatic resection to prevent bleeding complications, but expansion increases CVP. Deliberate lowering of the CVP during liver resection assists in bleeding control by decreasing the blood pressure gradient over which bleeding occurs during dissection.[2] Massicotte *et al*. showed that maintenance of a low CVP prior to the anhepatic phase, avoidance of plasma transfusions and restricting volume replacement during OLT was associated with a decrease in RBC transfusions during OLT.[16]

#### Cell salvage:

The use of cell salvage to collect and reinfuse shed, autologous blood is common practice in OLT, when high blood loss is anticipated.[2] Autologous blood transfusion reduces the risks associated with allogenic transfusions. Cell salvage is not indicated in the presence of sepsis and hepatocellular carcinoma (HCC).[2] Large volume transfusion of salvaged blood can cause postoperative hypofibrinogenemia, thrombocytopenia, prolonged prothrombin and partial thromboplastin time and elevated fibrin split products.[17] This cell saver induced coagulopathy can be prevented by simultaneous platelet and cryoprecipitate transfusions along with reinfusion of salvaged blood, as salvaged blood does not provide platelets and fibrinogen.[17,18] Saline washing of red cells increases sodium levels and decreases potassium and calcium levels. Hence, supplementation of potassium and calcium is done during cell salvage.

### Postoperative factors

Primary nonfunction of graft or delayed graft function

Failure of graft to function contributes to postoperative bleeding, causing coagulopathy.[2] Appropriate blood component therapy is given to these patients.

### Leaks at anastomosis

Leaks at vascular suture lines or bleeding from the cut surfaces at bowel anastomoses due to technical failures, result in postoperative bleeding which may require re-intervention and blood transfusions.[2]

### Graft versus-host disease

GVHD can occur from transfer of donor-derived passenger lymphocytes and manifests as hemolysis during 7-14 days after transplantation.[6] This is controlled by transfusion of donor-specific RBCs.[6]

Sepsis: Sepsis also results in multiple blood transfusions during the post-operative period.

### Thrombocytopenia

It causes bleeding which is associated with platelet consumption, platelet-associated IgM and IgA antibody production, sequestration, thrombin generation, viral infection and ABO-incompatible GVHD.[2] This further necessitates platelet transfusions.

## Drugs that Minimize Blood Loss

Antifibrinolytics: Increased fibrinolysis is observed in some patients during the second and third phases of OLT. Various antifibrinolytic agents like aprotinin (AP), tranexamic acid (TA) and epsilon amino caproic acid (EACA) have been used to counter this accelerated fibrinolysis.[2] Prophylactic inhibition of hyperfibrinolysis with the biological serine protease inhibitor AP or the synthetic lysine analog TA is common clinical practice in OLT.[19] Aprotinin has been shown to significantly decrease blood loss, transfusion of RBC units, FFPs, platelets and cryoprecipitate and duration of surgery.[20] It also has an effect on platelet function, anti inflammatory properties and the need for intraoperative ionotropes.[20,21] Tranexamic acid also decreases transfusion requirements in some but not all studies.[2]

Recombinant factor VIIa (rFVIIa) has been shown to improve hemostasis during liver transplantation.[5] A single dose of 80 mcg/kg rFVIIa significantly reduced transfusion requirements during OLT.[5,22] A study was done where rFVIIa was given as a bolus just before surgery to patients with preoperative risk of high intraoperative blood loss, including severe uncorrected coagulopathy.[2] There was immediate correction of coagulation after administration of rFVIIa and these patients were adjusted to normal risk group without an increased risk for thrombotic complications.[2]

Conjugated estrogen administered in a dose of 100 mg prior to surgery and just after graft perfusion has shown to decrease blood loss and transfusion requirement.[2] Frenette *et al*. reported statistical decrease in the use of blood products when conjugated estrogen was used instead of placebo during OLT.[23]

Clonidine, a centrally acting alpha2-adrenergic receptor agonist, significantly decreased transfusion and fluid requirements in a small prospective, randomized controlled trial.[2]

## Orthotopic liver transplantation without transfusion

The literature includes cases of OLT performed without transfusion of any blood products and OLT performed safely without additional blood products if blood loss is limited to 1,600-3,400 ml.[2] Reports have described OLT in Jehovah's witness patients (for religious reasons, Jehovah's witnesses refuse transfusion of blood products), who received no blood products.[2] Jabbour and colleagues continue to lead the field in performing OLT without the use of blood products. They reported favourable results in 27 consecutive patients who underwent transfusion-free liver transplantation.[2,3] This team used a combination of preoperative stimulation of red cell production using recombinant human erythropoietin and iron and intraoperative hemodilution, cell salvage, and tolerance to moderate anemia.[3] They reported successful use of rFVIIa in 10 patients, just prior to the incision.[3] Another team led by Olivier Detry reported successful transfusion-free liver transplantation in 9 Jehovah's witnesses.[24]

## Transfusion support

ABO grouping: Liver transplants must be ABO compatible, because of the complement-fixing effect of ABO antibodies on endothelial cells.[6] Efforts to cross the traditional ABO barrier fall into three categories: (i) neonatal recipients (ii) A_2_ organs (iii) fully ABO-incompatible organs.[7] Major ABO-incompatible liver transplants do not undergo hyperacute rejection, but their short-term graft survival is significantly reduced. These protocols utilize plasmapheresis, multiple immunosuppressive agents, and sometimes splenectomy.[7]

An error in ABO compatibility for organs can be life threatening for the patient. Hence, the United Network for Organ Sharing (UNOS) instituted new requirements for additional clerical verification of correct ABO types for transplant donors and recipients.[7] UNOS also proposed new policies requiring the performance of two separate ABO typings of each organ donor and recipient.[7]

## Selection of compatible blood components

### 

#### ABO blood-type selection[6]:

Group A or B patients: Group A or B are managed with type-specific blood and switched to group O blood, if necessary

#### Group AB patients:

Can receive RBCs of any group. ‘AB’ RBCs available through outdating should first be used, then switched to group ‘A’ RBCs. After transfusing 10 units of group ‘A’ RBCs, patient can be switched to group ‘A’ plasma.

#### Group O patients:

Only group ‘O’ RBCs can be used in these patients, they can receive any ABO group plasma.

#### Rho (D) selection[6,7]:

Rh-negative patients should be provided with D- RBCs to avoid sensitization to D. When transfusion requirements become excessive, D-women over child bearing age, and adult men may be switched to D + RBCs, provided anti-D is not detected before transfusion. Effort should be made to maintain D-women of child bearing age and children on D-blood, although the risk of D alloimmunization in OLT is low perhaps due to postoperative immunosuppression. Such patients should be given Rh immunoglobulin (RhIg) after administration of Rh-positive blood components. A case of severe anti-D mediated hemolysis resulting from liver transplantation in a O Rh + man who received an O Rh- liver allograft is reported.[25] The female O Rh – donor had alloantibodies against D,C, and K. This patient required two red blood cell exchanges and intermittent red cell transfusions negative for D, C, and K. A normalization of hemoglobin levels and decrease in serum bilirubin occurred after a splenectomy on postop Day 321.[25]

## Selection of red cell units in patients with clinically significant allo-antibodies

Clinically significant allo-antibodies are present in 6% of liver transplant patients, due to sensitization through previous blood transfusions.[6] To minimize the risk of hemolysis, these patients are managed by using antigen-negative units for the first 5-10 units, switching to antigen unscreened and/or partially matched units in the middle of the case, and then switching back to antigen-negative units for the last 5-10 units to prevent postoperative hemolysis.[6] This strategy requires close co-operation between anesthesiologist and transfusion services. An additional strategy would be to use preoperative plasmapheresis to remove clinically significant low-titer antibodies.[7]

Use of Autologous Blood: Pre-operative autologous blood deposit, perioperative isovolemic hemodilution, and reinfusion of salvaged blood by cell saver during surgery minimizes allogenic transfusions.[7]

## Specialised blood components

CMV reduced risk components: As a result of immunosuppression, liver transplant recipients are more susceptible to some transfusion complications such as cytomegalovirus (CMV) infection.[6] Steps should be taken to prevent transmission of CMV through blood transfusions to liver transplant recipients who have not been previously infected (i.e., CMV-seronegative recipients of seronegative donors).[26] Prevention can be achieved by leucocyte reduction of the cellular components, by selection of blood from CMV-seronegative donors, or both.[26]

Leucocyte-reduced blood components: Transfusions before, during, and after transplant surgery should be leucocyte reduced to reduce the risk of developing HLA alloimmunization.[26] If HLA antibodies develop in patients waiting for an organ transplant, the risk of rejecting the transplanted organ increases and the chance of finding a compatible organ decreases.[26]

Irradiated blood components: Graft versus-host disease (GVHD) is a rare complication of organ transplantation and is almost always due to donor organ. These few cases do not support a policy of routine irradiation of cellular blood components for solid organ transplant recipients.[6]

‘Male only plasma’: The strategy to issue plasma from male donors for transfusion is an effort to reduce the incidence of Transfusion Related Acute Lung Injury (TRALI), as plasma components linked to female donors (parous women) with neutrophilic antibodies are responsible for the majority of probable TRALI cases.[27]

Minor ABO-incompatible transplants: When group O allografts are transplanted to nongroup-O patients/nongroup-AB allografts are transplanted to group AB patients, donor passenger lymphocytes are transferred with the organ at the time of transplantation and produce alloantibodies against host antigens in the recipient causing delayed hemolysis.[7] Clinically significant hemolysis caused by anti-Jk(a), produced by passenger lymphocytes transferred from the donor's liver to the recipient has been reported.[6] These donor derived antibodies(DDAb) develop 7-14 days after transplantation and is heralded by the development of positive direct antiglobulin test (DAT).[6] Serum antibody is predominantly IgG. DDAb are short lived antibodies that persist for 2-3 weeks. Ramsey reported the incidence of DDAb and hemolysis in OLT as 40% and 29%.[6] Hemolysis is usually mild and self limiting. This is more likely in group A recipients of group O livers who receive cyclosporin or tacrolimus immunosuppression.[6] In most cases the hemolysis associated with DDAb is mild and can be treated with transfusions.[6] Several cases of hemolysis-induced acute renal failure have been reported. In fulminant cases, red cell exchange with donor ABO group or group O red cells, or plasma exchange, has been performed.[6]

### Transfusion protocol in minor ABO mismatched liver transplants

1. Transfusing exclusively donor group red blood cells(RBCs) from the beginning of surgery.[6,7]
2. Transfusing recipient ABO group of red cells at the beginning of surgery, but switching to donor ABO group of red cells after early reperfusion phase up to six weeks in postoperative period. This replaces susceptible red cells with cells that will not be hemolyzed. Plasma products are transfused based on recipient ABO group to reduce the risk of hemolysis by providing soluble ABO antigen capable of neutralizing DDAb. After six weeks, if DAT and anti-A/anti-B antibodies are negative, red cells are switched over to recipient's ABO group.[6,7]
3. Transfusing recipient group RBCs, but performing postoperative DAT and switching to donor group RBCs if DAT is positive.[6,7]
4. Transfusing recipient group RBCs until incompatibility is noted in a crossmatch.[6,7]

## Transfusion Protocol During Liver Transplantation

### 

#### Clinical protocol[4]:

RBCs, fresh frozen plasm (FFP), and crystalloid are given in the rapid transfusion device and in the intravenous lines as per following ratio:

2 units of RBCs (banked + salvaged) + 1 unit of FFP 1 + 200 ml of crystalloid. This mixture will generate hematocrit (Hct) >30%, prothrombin time (PT) in the range of 14-18 seconds, and fibrinogen >100 mg/dl.

Each salvaged unit counts as a unit of RBCs. Switch from crystalloid to a mixture of crystalloid and 5% albumin, if the patient requires a cumulative total of 125 ml/kg crystalloid from all sources.

#### FFP:

In the presence of active fibrinolysis and fibrinogen <80 mg/dl, the PT/aPTT will overestimate the degree of hemodilution. Mild to moderate prolongations of the PT need not be corrected. In the absence of active fibrinolysis or a surgical bleeder, an INR <2.5 is adequate.

#### Platelet concentrate:

The platelet count will halve from dilution with each blood volume transfused. In nonrefractory patients, maintain platelet count >75 × 10^3^/mm^3^ for patients without severe splenomegaly and >50 × 10^3^/mm^3^ for those with splenomegaly. For refractory patients, do not transfuse for numbers.

#### Cryoprecipitate and aminocaproic acid:

Most patients do not require cryoprecipitate. For fibrinolysis (fibrinogen < 100 mg/dl) with bleeding, give 5 g intravenous aminocaproic acid infusion. If lysis continues after 1 hour, reload aminocaproic acid, and begin drip of 1 g/h. Transfuse 10 units cryoprecipitate.

## Another protocol

Criteria for replacement of blood products are as follows: Administration of red cell units to maintain Hemoglobin (Hb) levels at 8 gm%; FFP (1 unit/20 kg) in case of hemorrhage associated with International normalized ratio (INR) >2.0; apheresed platelets to maintain platelet count >50 × 10^3^/mm^3^ (>80 × 10^3^/mm^3^ in massive transfusion) and cryoprecipitate (1 unit/10 kg) for fibrinogen levels <100 mg/dl in case of ongoing bleed (<120 mg/dl in massive transfusion).[14,28]

Hourly laboratory monitoring of hemoglobin (Hb), platelet count (PT), and fibrinogen are done throughout the surgery. Cell saver is used to salvage blood for autologous transfusion. In case of massive blood loss, rapid infusion pump is used to facilitate fluid resuscitation and transfusions are given in a blood warmer. Air-warming blankets are used to maintain body temperature.

In OLT, there is institutional variability in transfusion practice, which mandates reassessment of the rational use of blood products.[29]

## Massive transfusion

When transfusions exceed more than one blood volume within 24 hours, it is considered as massive transfusion.[18]

Patients are grouped into three categories according to intraoperative blood volume transfused:[30]

Group A: 1.5 or less blood volume transfused

Group B: >1.5 and <3 volumes used

Group C: 3 or more volumes given

### Complications of massive transfusions

Dilutional coagulopathy: Volume replacement with crystalloids and colloids and temperature control are initial resuscitation measures. This is followed by transfusion of red cells, coagulation factors and platelets, and DIC requires treatment.[31]

Dilutional thrombocytopenia: It has been shown that at least 1.5 times blood volume must be replaced for this to become a clinical problem. It can occur following smaller transfusions, if DIC occurs or there is preexisting thrombocytopenia.[31]

DIC: There is association of hemostatic defects related to the exaggerated generation of thrombin and fibrin and the excessive consumption of platelets and coagulation factors which is one of the causes for massive bleed in postanhepatic phase. DIC has been correlated with ischemic damage of the graft liver.[2]

Citrate toxicity and hypocalcemia: Each unit of blood contains 3 grams of citrate, which binds calcium. Healthy liver metabolises 3 grams of citrate every 5 minutes. Transfusion at rates higher than one unit every 5 minutes or impaired liver function leads to citrate toxicity, hypocalcemia, and hypomagnesemia. Calcium should be given if there is biochemical, clinical or ECG evidence of hypocalcemia.[31]

Hyperkalemia: Hyperkalemia is not a problem unless very large amounts of blood are given quickly. Hypokalemia is more common as red cells begin active metabolism and intracellular uptake of potassium restarts.[31]

Acid/base disturbances: Lactic acid levels in a blood pack give an acid load of up to 30-40 mmol/l. This along with citric acid is usually metabolized rapidly. Citrate is metabolized to bicarbonate, and a profound metabolic alkalosis may ensue.[31]

Hypothermia: Hypothermia leads to reduction in citrate and lactate metabolism (leading to hypocalcemia and metabolic acidosis), increase in affinity of Hb for oxygen, impairment of red cell deformability, platelet dysfunction and an increased tendency for cardiac dysrhythmias.[31]

Immunosuppression: Large volumes of allogenic blood transfusion results in the infusion into the recipient of large amounts of foreign antigens in both soluble and cell-associated forms, the persistence of these antigens in the circulation of the recipient is considered to result in immune downregulation.[14]

Immunomodulation: Numerous retrospective and prospective studies have observed a significant association between allogenic blood transfusion and immune suppression, including graft survival, recurrence after surgery of a variety of malignancies, impaired cell-mediated cell and natural killer cell activity, and deterioration in liver regeneration.[14]

Sepsis: Large transfusion requirements, i.e., excessive blood loss during OLT are correlated with reduced graft survival (graft dysfunction) and increased septic episodes, prolonged intensive care unit stay and morbidity and mortality.[14]

Reintervention: Intraoperative red cell transfusion requirement was the main determinant of early in-hospital surgical reintervention after liver transplantation.[32]

Acute respiratory distress syndrome: ARDS is a serious multifactorial complication after OLT with a high mortality and fatality. The most likely cause is fluid overload from crystalloid liquid infusion or massive transfusion. Other factors such as TRALI and reperfusion syndrome of the newly implanted liver may also contribute.[33]

Intracerebral hemorrhage: Intracerebral hemorrhage is one of the most disastrous complication after OLT.[34] Intraoperative hypotension, massive intraoperative transfusion and coagulopathy may be correlated with this complication.[34]

## Conclusion

### Multi disciplinary approach

Proper patient selection, better preservation of graft, short CIT, meticulous surgical technique, skilled anesthesia practice, intraoperative auto transfusion, use of antifibrinolytic drugs and rFVIIa during surgery, use of TEG, and administration of appropriate components, optimal use of available new technologies, and drugs, minimizes transfusions during OLT. Efforts to reduce intraoperative bleeding leading to limitation of the amount of blood used are therefore desirable not only to improve results but also to control costs, preserve the blood pool for other emergency or routine surgeries and to decrease the program's dependence on blood availability. Goal should be to develop strategies towards a transfusion free environment.
